# Endoscopic Ultrasound-Guided Fine Needle Aspiration (EUS-FNA) Diagnosis of Recurrent Anal Cancer After Chemoradiation and Negative Forceps Biopsies: A Case Report

**DOI:** 10.4137/cmo.s993

**Published:** 2009-04-28

**Authors:** Julia LeBlanc, Pradermchai Kongkam

**Affiliations:** Indiana University Medical Center, Division of Gastroenterology and Hepatology.

## Abstract

A 69-year-old woman with a history of uT2 N0 post-treated anal squamous cell cancer (SCC) presented for EUS for perianal pain. Two months prior, a digital rectal examination was significant for an indurated lesion on the left lateral rectal wall just proximal to the dentate line. A sigmoidoscopy revealed mild narrowing of the anal canal and an ulcerated friable mucosa in the same area. A biopsy demonstrated ulceration without malignancy. EUS showed a hypoechoic, non-circumferential, left-sided distal rectal mass. EUS-FNA was performed. Cytology demonstrated poorly differentiated SCC. This was confirmed by subsequent surgical resection. While endoscopic biopsy of suspected anal recurrences is usually sufficient, histologic or cytologic confirmation are necessary, as radiation-induced changes are difficult to differentiate from tumor recurrence. This case demonstrates that EUS-FNA is useful in surveillance of anal SCC when there is a high clinical suspicion of recurrence.

## Introduction

Anal squamous cell cancer (SCC) is an uncommon disease^1^ that responds well to chemotherapy and radiation. There is no established standard for post treatment surveillance. Perianal tissue inflammation occurring after radiation makes differentiation between recurrent cancer and radiation-induced injury difficult.^2^ We herein report a case of a woman with a history of anal SCC status post chemoradiation who presented with perianal pain and ulceration. She underwent lower endoscopy of the anal canal and rectum with negative biopsies on two separate occasions. She was referred for endoscopic ultrasound (EUS) with possible fine needle aspiration (EUS-FNA) of the anal canal.

## Case Report

A 69-year-old white woman with a history significant for hypothyroidism and neck immobilization was diagnosed with uT2 N0 anal squamous cell carcinoma (SCC) of the left lateral anal canal. She initially presented with perianal pain. A pelvic CT scan performed at the time of diagnosis was unremarkable. She underwent chemotherapy and external beam radiation. Her chemotherapy regimen consisted of mitomycin C and oral capcitebine. Following chemotherapy and radiation, a repeat lower endoscopy with an anal biopsy was performed which revealed persistent SCC in situ. She then underwent a second course of chemotherapy and radiation.

Four months later during follow up an indurated lesion on the left lateral rectal wall adjacent to the dentate line and an anal stricture was appreciated on digital rectal examination. A gastroenterologist performed a sigmoidoscopy which was significant for a left lateral rectal ulcer and an anal canal stricture. Biopsies were taken from the rectal ulcer and anal dilation was performed. The biopsies were negative for malignancy. One month later, the patient presented with rectal bleeding and severe perianal pain exacerbated with defecation and sitting. A second sigmoidoscopy revealed mild narrowing of the anal canal and ulcerated friable mucosa in the left lateral distal rectum involving the anal canal. Repeat biopsies of the ulcerated mucosa in the rectum and anal canal demonstrated ulceration without malignancy.

One month later and 6 months after completing her second course of chemotherapy and radiation, she presented for EUS of the anal canal. Prior to the EUS, the gastroenterologist performed a digital rectal examination. The examination was significant for exquisite tenderness and induration of the left lateral anal canal and distal rectal wall extending approximately 2 cm from the anal verge.

**Figure 1.**
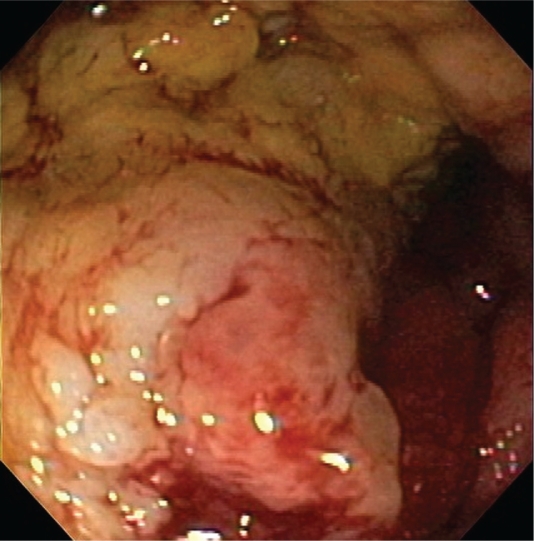
Radial EUS revealed a hypo-echoic, left-sided distal rectal mass.

**Figure 2.**
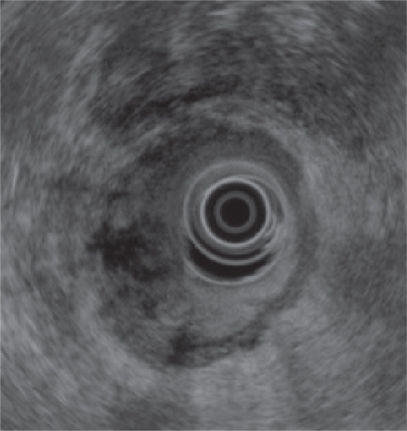
The mass was non-circumferential with poorly defined borders and encompassed approximately 50% of the circumference of the distal rectum.

**Figure 3.**
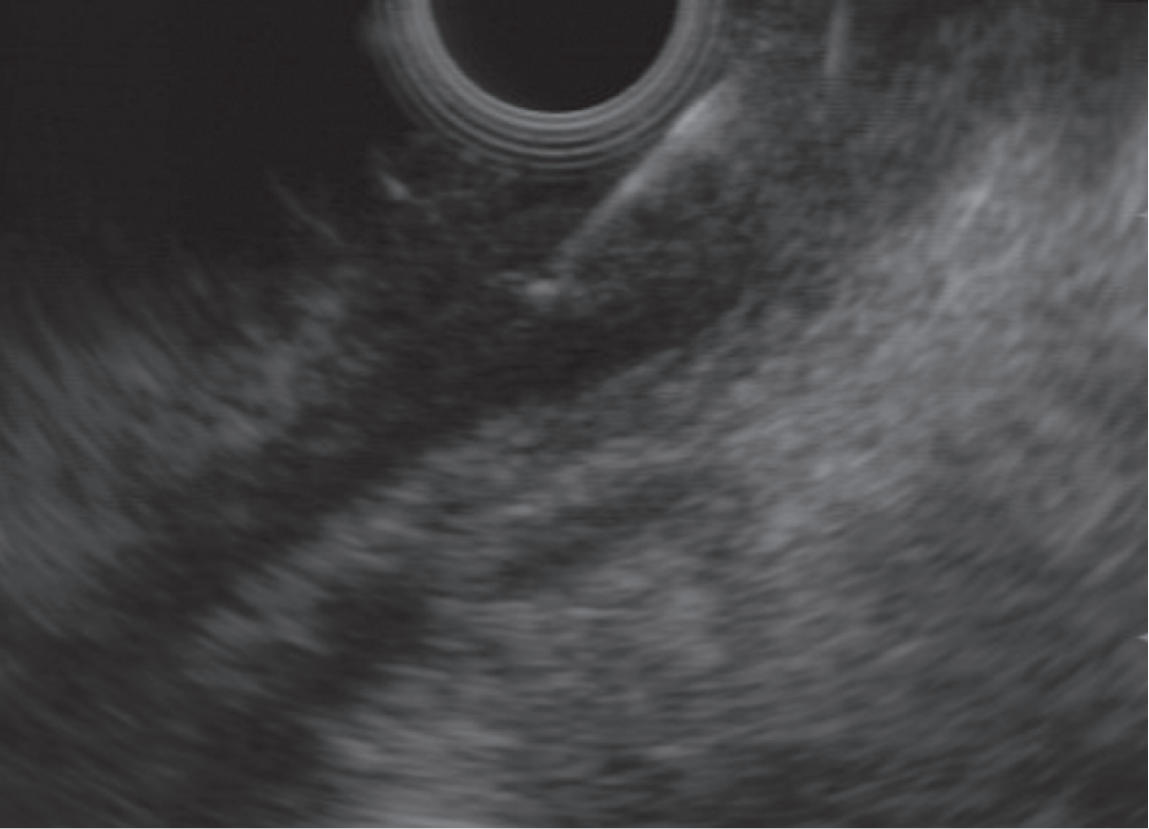
EUS-FNA of the tumor recurrence.

**Figure 4.**
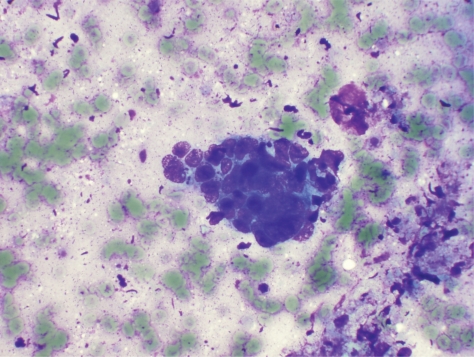
EUS-FNA aspirate (Hematoxylin and Eosin staining, 40 X) revealing poorly differentiated squamous cell carcinoma of the anal canal. Peripheral nuclear palisading cells are seen.

On radial EUS (Olympus GF-UM160, Center Valley, PA) examination a hypoechoic, left-sided distal rectal mass was seen (Fig. 1). The mass was non-circumferential with poorly defined borders. It encompassed approximately 50% of the circumference of the distal rectum (Fig. 2). The mass measured 18 mm in maximal thickness. Endoscopic ultrasound-guided fine needle 1 (EUS-FNA) of this abnormal area was performed (Fig. 3). Seven passes were taken with a 22-gauge needle (Echotip, Wilson Cook, Winston Salem, NC) using a transrectal approach. On preliminary on-site cytologic evaluation, a few groups of atypical cells with inflammation and necrosis were observed. The patient received a 1.5 g of ampicillin/ulbactam intravenously after the procedure and amoxicillin/clavulanic acid 500 mg orally three times a day for seven days. Final cytology results demonstrated poorly differentiated SCC (Fig. 4).

**Figure 5.**
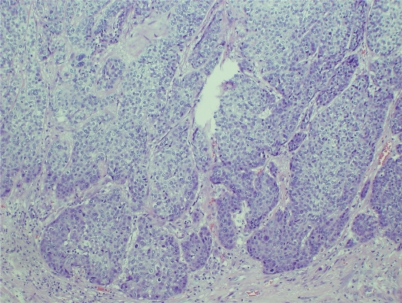
Surgical histology (Hematoxylin and Eosin staining, 40 X) revealing poorly differentiated squamous cell carcinoma.

The patient underwent abdominal perineal resection and colostomy. The posterior wall of the vagina, anus, and rectum were involved with poorly differentiated SCC (Fig. 5). The tumor extended to the subcutaneous tissue of the anus, with extensive perineural invasion. There was no evidence of lymphovascular invasion. Eight lymph nodes were negative for malignancy.

## Discussion

Endoscopic ultrasound (EUS) provides accurate TNM staging and has been used to determine clinical responsiveness after treatment.^3^ While endoscopic biopsies of suspected anal recurrences are usually sufficient, histologic or cytologic confirmation is necessary, as radiation-induced changes are difficult to differentiate from tumor recurrence. Symptoms and signs such as rectal bleeding, pain, skin changes, stricture, and proctitis may result from local recurrent disease or radiation-induced proctitis.^4^ Clinical history and physical examination with careful rectal examination are mandatory. Endoscopic mucosal biopsy is a standard tool for histological diagnosis but may also cause perianal or rectal pain and bleeding.^5^ Hence, endoscopic surveillance is usually performed at least 8 weeks after chemoradiation.^5^ Computed tomography (CT) or EUS without FNA cannot reliably differentiate between tumor recurrence and radiation-induced changes in the gastrointestinal wall.^6^,^7^

This case demonstrates the utility of EUS-FNA after non-diagnostic endoscopic biopsies were taken. The EUS-FNA cytopathology result was not able to differentiate between keratinizing and non-keratinizing SCC. However, given similar biology, prognosis and treatment, this differentiation is not necessarily required for management of anal SCC.^8^,^9^ Certainly, if the preliminary EUS-FNA had been negative an alternative would be to obtain an EUS-guided core biopsy (19 gauge) of the perianal canal for histologic assessment. This method acquires more tissue for histologic evaluation. In this particular case, had the EUS-FNA had been negative, the sonographic images of an irregular tumor combined with the clinical presentation would be sufficient to send this patient to surgery for resection, given this patient’s debilitating pain. However, sonographic imaging of suspected recurrence in the gastrointestinal wall should be confirmed cytologically or histologically in the absence of symptoms to suggest recurrence. Based on our experience, the hardness/stiffness of the target site and gross appearance of the aspirate are poor predictors of tumor recurrence in the gastrointestinal wall. We are not aware of any other features of the EUS-FNA procedure that would be predictive of recurrence in the gastrointestinal wall after chemotherapy and radiation.

To our knowledge, this is the only report of EUS-FNA used to diagnose post-treatment anal cancer recurrence after negative cold forceps biopsies. Our case demonstrates that EUS-FNA may be useful in surveillance of anal SCC following treatment when there is a high clinical suspicion of recurrence. Further prospective study of EUS surveillance of anal SCC recurrence is warranted in select patients.
